# Inbreeding Depression in Wild Mammals: Genomic Mechanisms and Conservation Consequences

**DOI:** 10.3390/ani16203268

**Published:** 2026-10-16

**Authors:** Ayesha Anwar, Tabris Yik To Chung, Chendi Zhu, Henry Chun Lok Tsui, Brian Chin Wing Kot

**Affiliations:** 1 Department of Infectious Diseases and Public Health, Jockey Club College of Veterinary Medicine and Life Sciences, City University of Hong Kong, Kowloon Tong, Hong Kong; aanwar5-c@my.cityu.edu.hk; 2 Department of Chemistry, College of Science, City University of Hong Kong, Kowloon Tong, Hong Kong; ytchung@cityu.edu.hk (T.Y.T.C.); chendzhu@cityu.edu.hk (C.Z.); cltsui9@cityu.edu.hk (H.C.L.T.)

**Keywords:** inbreeding depression, wild mammals, conservation genomics, runs of homozygosity, autozygosity, mutation load, purging, genetic rescue

## Abstract

Inbreeding depression is a widespread but highly variable threat to wild mammal populations, especially small, isolated, or fragmented ones. Genomic tools can now estimate individual inbreeding and mutation load more precisely than pedigrees or a few genetic markers. They show that similar levels of autozygosity can have very different fitness consequences. In this structured narrative review, we examine how runs of homozygosity, genomic inbreeding coefficients, and mutation load relate to inbreeding depression in wild mammals. We distinguish inbreeding (a process), autozygosity (its genomic consequence), low genetic diversity, and inbreeding depression (a fitness outcome), and we stress that high autozygosity alone does not demonstrate fitness costs. We compare land and marine case studies in which high autozygosity is associated with reduced reproduction, survival, and disease resistance, with cases in which purging or demographic history may have limited the decline in fitness. We consider how environmental stress, methodological differences, and confounding factors affect the observed inbreeding–fitness relationships. We then discuss how genomic estimates can inform conservation decisions, including the limits of genetic rescue, and we identify research priorities such as long-term studies that combine genomic, demographic, and ecological data. The review offers a framework for interpreting genomic inbreeding measures cautiously in conservation planning.

## Simple Summary

Small and isolated wild mammal populations often suffer reduced survival and reproduction because close relatives mate and harmful genetic variants are expressed. Modern genetic tools now measure inbreeding more precisely than older pedigree methods. This review explains how these genomic measures can help identify when inbreeding is associated with serious fitness problems and when populations appear buffered by past natural selection or demographic history. We summarise evidence from terrestrial and marine mammals and discuss how genetic information can guide practical actions such as managed gene flow (genetic rescue), while highlighting the limits of current evidence. These insights can help conservationists judge when and how to intervene so that threatened mammal populations remain viable in a changing world.

## 1. Introduction

Many wild populations are declining due to human activities, and their genetic diversity may be declining as well [1]. A meta-analysis of 91 species conservatively estimated a 5.4–6.5% loss of within-population genetic diversity in wild organisms since the Industrial Revolution [1]. Habitat fragmentation reduces biodiversity and impairs ecosystem functions, with effects greatest in the smallest and most isolated fragments [2]. Mammals are among the affected groups, from polar bears in the Barents Sea [3] to the Fennoscandian Arctic fox [4]. In small, isolated populations, inbreeding cannot be avoided, and it can substantially reduce fitness [5]. Inbreeding depression occurs in wild animal and plant populations [6]. Its strength varies widely. It depends on the genetic make-up of the species or population and on how genotypes interact with the environment [7]. It also differs among taxa, populations, and environments [8]. Genomic approaches can now measure individual inbreeding with high precision [9,10] and can estimate inbreeding depression more precisely than pedigrees [10]. The number and length of runs of homozygosity (ROH) also reflect an individual’s demographic history [11].

Inbreeding has several interpretations [12], so we first define the main terms and show how they relate to one another. Inbreeding is the mating of related individuals [13]. For an individual, it is quantified as the proportion of the genome that is identical by descent (IBD), measured relative to a reference base population [12]. The genomic consequence of this process is autozygosity, the realized proportion of an individual’s genome that is IBD. It can be estimated from pedigrees (*F*_p_) or from genomic data, for example, as the fraction of the genome in ROH [9]. Autozygosity (identical by descent) should not be confused with simple homozygosity (identical by state), which does not always mean shared ancestry. Two alleles are identical by state (IBS) when they are the same, whether or not they share a recent common ancestor [14]. This matters because ROH-based analyses implicitly assume that homozygous runs that are IBS are also IBD [14]. A second distinction is between autozygosity and genetic diversity, which reflects long-term population history. Populations that remain small for many generations can have exceptionally low genome-wide diversity [15,16], so low diversity is not the same as recent inbreeding. Another related concept is genetic load, the reduction in a population’s mean fitness caused by genetic variation. Genetic load comprises a realized component, which reduces fitness in the current generation, and a masked component, formed by recessive deleterious mutations whose effects may be expressed in future generations, for example, through inbreeding [17]. Finally, inbreeding depression is the fitness outcome of inbreeding: the decline in survival and reproduction that results from it [13]. Because autozygosity and low diversity describe the genome, whereas inbreeding depression describes fitness, high autozygosity or low diversity does not, by itself, indicate inbreeding depression. That must be shown by relating inbreeding to fitness-related traits.

Small populations increasingly face isolation and inbreeding, and genetic management has become a vital tool [18]. In a study of scimitar-horned oryx, for example, unmanaged groups were enriched for long ROH and carried a higher burden of homozygous deleterious genotypes than managed groups [18]. Genomic studies of wild mammals cover both land and sea. On land, long-term studies of wild red deer and Soay sheep have linked genomic inbreeding to fitness [19,20]. Marine mammals, however, deserve particular attention for three reasons. First, some have slow life histories and delayed reproduction. Bowhead whales, for example, reach sexual maturity at around 25 years and can live for more than 100 years [21], while captive studies of endangered Yangtzu finless porpoise demonstrate how environmental cues such as photoperiod regular testicular dynamics and seasonal sexual behavior. Second, some live in socially structured populations. In matrilineal whales, culturally transmitted behaviour could reduce mitochondrial genetic variation [22], and cultural traditions play a role in ecological niche partitioning in cetaceans [23]. Third, their biology limits the management options available. Genomic studies of marine mammals also show wide variation in inbreeding. Some populations are extremely autozygous [16]. Others carry a low burden of segregating deleterious variation [24] or are inferred to have purged much of their genetic load during an extreme bottleneck [25].

Inbreeding depression has been studied extensively. Other work has addressed genetic load [17], purging [26,27], ROH inference in wild populations [28], and the link between ROH and inbreeding depression or fitness [29,30]. An earlier review of inbreeding in natural mammal populations [31] predates many of the whole-genome studies discussed here. What is still missing is a review of wild mammals that brings together genomic inbreeding measures, fitness evidence, and management options. A study should also compare land and marine species, for which the evidence and the management options differ. This review addresses that gap. We have three aims. The first is to separate inbreeding, autozygosity, low diversity, mutation load, and inbreeding depression when interpreting case studies. The second is to examine why similar levels of autozygosity are associated with different fitness outcomes, considering demographic history, purging, environmental context, and methodological differences. The third is to assess what genomic evidence can and cannot currently contribute to conservation decisions, including genetic rescue, for species in which translocation is not feasible.

## 2. Methods

This article is a structured narrative review. It is not a systematic review or meta-analysis. It does not attempt exhaustive retrieval or statistical pooling of results, so a PRISMA flow diagram is not presented. We searched PubMed, Web of Science (Core Collection), and Scopus between September 2025 and April 2026, and we considered peer-reviewed papers available at the time of the final search in April 2026. Keywords were combined with Boolean operators and searched in titles, abstracts, and keywords (Title/Abstract in PubMed, Topic in Web of Science, and TITLE-ABS-KEY in Scopus). Representative search terms included (“inbreeding depression”) AND (“wild mammals” OR “wildlife”); (“runs of homozygosity” OR “ROH”) AND (“conservation genomics” OR “autozygosity”); and (“mutation load” OR “purging”) AND (“genetic rescue”) AND (“mammal” OR “cetacean”). The exact strings entered in each database were not archived at the time of screening. We also found studies in the reference lists of included articles and recent reviews (backward citation chaining), as well as through our own knowledge of the field. One author (A.A.) screened titles and abstracts and then assessed the full texts of potentially relevant articles against the eligibility criteria. All primary empirical studies of wild mammals that are discussed are catalogued by taxonomic group, genomic measure, and fitness outcome in Supplementary Table S1.

We included studies that provided empirical, theoretical, or simulation-based evidence on inbreeding in mammals; genomic measures of autozygosity (e.g., ROH and single-nucleotide polymorphism (SNP)-based coefficients); mutation load and purging; the fitness consequences of inbreeding; or genetic management and other conservation interventions. We excluded studies of domestic livestock that were not relevant to conservation, studies of non-mammalian species, and grey literature. A few foundational methodological, theoretical, or experimental studies on other taxa were retained where needed to define concepts or estimators. We did not log the number of records retrieved and excluded at each stage. Instead, all primary empirical studies of wild mammals discussed here are listed in Table S1 by taxonomic group, study system, inbreeding measure, and fitness outcome. As a review, this article has no Results section. Section 3 covers the genetic basis of inbreeding depression and the methods used to estimate inbreeding. Section 4 and Section 5 review the evidence on fitness consequences and why they vary. Section 6 and Section 7 cover conservation implications and future research.

## 3. Genetic Mechanisms of Inbreeding Depression and Approaches to Estimating Inbreeding

### 3.1. Mutation Load, Purging, and Inbreeding Depression

Inbreeding depression is thought to be caused mainly by recessive deleterious mutations [6]. Two hypotheses have been proposed to explain how inbreeding reduces fitness. Under the partial-dominance hypothesis, inbreeding increases the frequency of homozygotes for deleterious recessive alleles, whereas under the overdominance hypothesis, it reduces the frequency of superior heterozygotes [5]. Partial dominance, therefore, fits the current evidence better, and an experimental test of both hypotheses in the sand cricket *Gryllus firmus* also supported partial dominance [32]. This distinction matters for conservation because of purging. Purging is selection induced by inbreeding against homozygotes for (partially) recessive deleterious alleles, and it becomes more efficient as population size increases [33]. In small populations, purging through drift can remove only highly recessive mutations [34].

Genetic load has two components: the realized load and the masked load [17]. Large populations can carry high levels of recessive, strongly deleterious variants that are hidden from selection in the heterozygous state [35]. As populations decline and recover, purging and genetic drift both alter the distribution of deleterious mutations [27]. Increased homozygosity exposes strongly deleterious recessive variants to selection. This can remove them (purging) and reduce the burden of strongly deleterious recessive alleles [15,36]. At the same time, bottlenecks can relax selection, so mildly deleterious mutations can accumulate [36]. Drift can also fix deleterious alleles, which increases the realized load [25]. The extent to which purging reduces inbreeding depression depends on dominance and population size [26]. Purging should not be expected to eliminate inbreeding depression because substantial fitness costs persist even in populations that have been small for a long time [30].

### 3.2. Pedigree, Heterozygosity and Single-Nucleotide Polymorphism (SNP)-Based Estimates

The inbreeding coefficient of an individual is the probability that it has two alleles at a locus that are identical by descent [8,13]. Pedigrees have limits as they are hard to obtain, can be unreliable, and seldom capture inbreeding from common ancestors more than a few generations back [37]. In simulations, the realized proportion of the genome that is IBD was more closely correlated with genomic measures based on thousands of SNPs than with the pedigree coefficient [9]. Even when pedigrees spanned 20 generations, the pedigree coefficient explained <80% of the variation in this proportion on average [9]. Pedigrees can also miss inbreeding depression in adult fitness. In wild red deer, genomic coefficients detected inbreeding depression in annual breeding success of both sexes. Still, pedigree-based coefficients detected it only in juvenile survival and in none of the adult fitness components [19]. Marker-based measures are also limited, as marker heterozygosity was correlated with inbreeding only under narrow conditions, when inbreeding events were both frequent and severe [38]. Heterozygosity-fitness correlations can be used to estimate the effect of inbreeding, but these estimates are necessarily imprecise, particularly when inbreeding is weak [39]. Dense SNP data allow other estimators. A comparison using simulated data showed that, in populations with small effective size, SNP-by-SNP estimators that placed weight on rare alleles substantially overestimated inbreeding depression. In contrast, in populations with a small effective size, ROH-based estimates were very accurate [40]. Finally, the choice of statistical model also matters, particularly in structured populations [41].

### 3.3. Runs of Homozygosity (ROH)

Runs of homozygosity are long homozygous segments of a chromosome that arise when identical haplotypes are inherited from each parent [11]. They reflect IBD patterns and can be used to measure individual inbreeding [28]. The genomic inbreeding coefficient *F*_ROH_ is the proportion of the autosomal genome that lies in ROH above a specified minimum length [42]. It is calculated as *F*_ROH_ = ΣL_ROH_/L_AUTO_, where ΣL_ROH_ is the total length of an individual’s ROH above the minimum length, and L_AUTO_ is the length of the autosomal genome covered by the SNPs, excluding centromeres.

Genomic and pedigree estimates of inbreeding can agree closely in well-studied populations. In a European human population from Orkney, *F*_ROH_ based on ROH of at least 1.5 Mb correlated strongly with pedigree-based inbreeding coefficients (r = 0.86) [42]. In Scandinavian wolves, inbreeding estimated from an extensive pedigree also correlated strongly with realized inbreeding measured across the whole genome (r^2^ = 0.86) [43].

**Figure 1:**
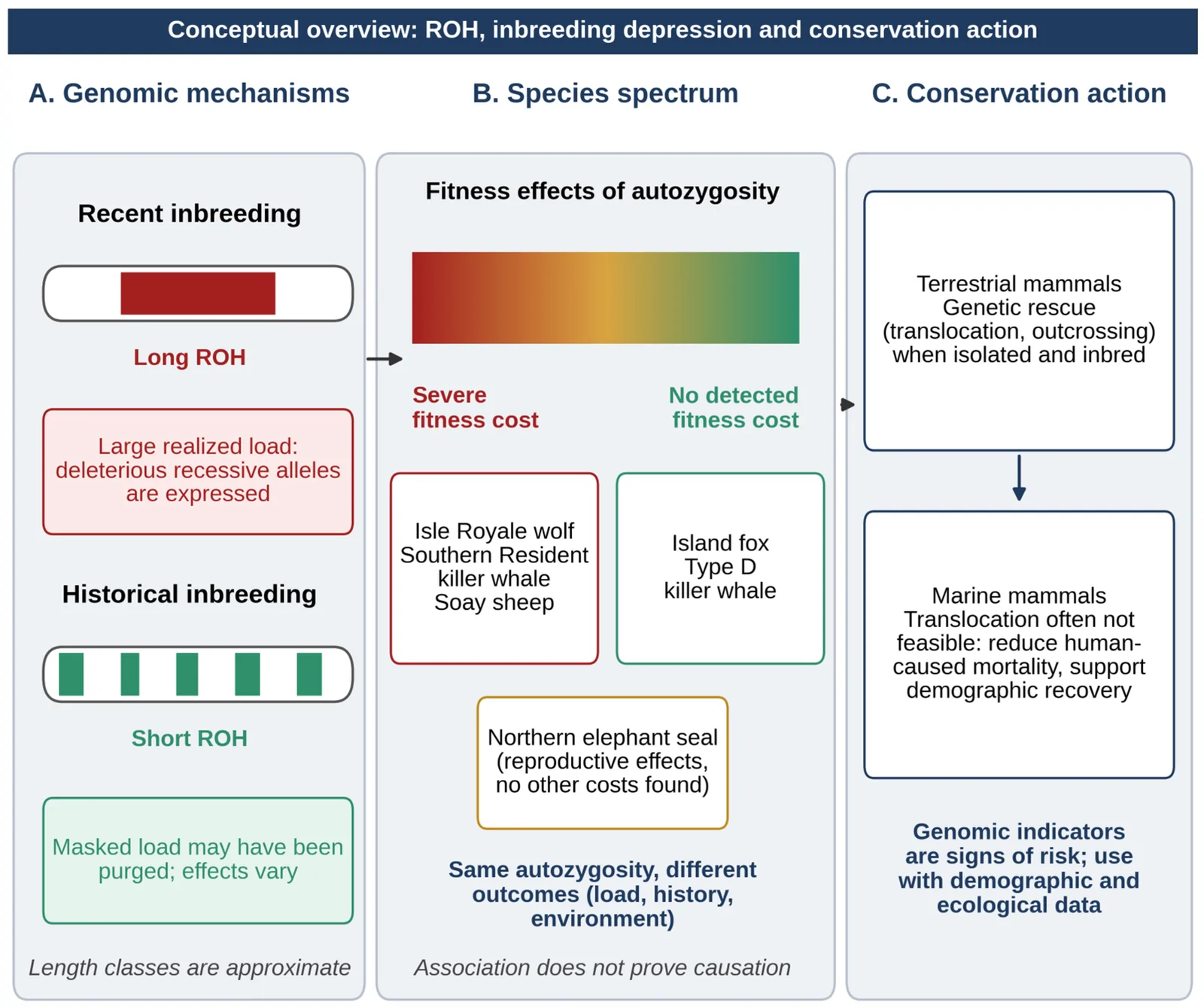
Runs of homozygosity (ROH) as an indicator of inbreeding history and fitness in wild mammals. The schematic is conceptual: ROH length classes and the corresponding numbers of generations are approximate and depend on recombination rate, marker density, and ROH-calling parameters; an association between ROH and fitness does not, by itself, establish a causal effect.

ROH length also indicates how long ago the shared ancestry occurred. Long ROH come from recent common ancestors, whereas short ROH come from more distant ones [11]. In Scandinavian wolves, most individual inbreeding was due to long IBD segments (>5 cM) inherited from ancestors no more than 10 generations back [43]. Killer whale genomes show both patterns. Individuals from populations that had experienced ancestral declines in effective population size (*N*_e_) carried hundreds of short ROH (<1 Mb), which indicates high background relatedness from shared ancestry far back in the pedigree [44]. Longer ROH (>1.5 Mb), which are younger, occurred in low-latitude populations and in populations of known conservation concern [44]. Figure 1 illustrates how ROH length relates to inbreeding history.

However, ROH of up to 4 Mb are common even in demonstrably outbred humans with no shared ancestors in at least five generations [42]. Length thresholds are therefore approximate rather than universal. Several other things affect ROH length and number. The processes that determine IBD are highly variable [45]. ROH are unevenly distributed across the genome and are somewhat more common in regions of low recombination, and their number and length reflect demographic history and population size [11]. ROH estimates also depend heavily on SNP density, sequencing depth, missing data, and the software settings used [46]. Despite these limits, a strong link between long ROH and inbreeding depression has been reported [29]. Comprehensive ROH-based approaches that include all ROH lengths also show promise as conservation monitoring tools [30].

These methodological dependencies matter when studies are compared. In a methodological study of a threatened bird that compared genomic data at different resolutions, low-density datasets tended to miss ROH in some genomic regions and to overestimate ROH length in others [46]. Across taxa, the detection methodology for ROHs significantly influenced the strength of ROH–fitness associations [30]. Comparisons of *F*_ROH_ between studies should therefore be made with caution.

## 4. Fitness Consequences of Inbreeding in Wild Mammals

Inbreeding depression affects many fitness traits, but its expression varies among populations, traits, and ecological contexts. The effects reported most often involve reproduction, survival, and disease resistance. Most of the evidence below consists of associations between individual inbreeding and fitness-related traits, so causal interpretation requires caution.

### 4.1. Reproductive Impairment

Reduced fertility is one of the two defining components of inbreeding depression, together with reduced survival [6]. The evidence comes from a few well-studied species, and the results differ between them. A classic example is the cheetah. In this species, an extreme lack of genetic variation, likely due to a severe population bottleneck in the recent past, has been associated with difficulty in captive breeding, high juvenile mortality, and a high frequency of sperm abnormalities [47]. However, a later field study found that most cub deaths in the wild were caused by predation (73.2% of deaths, most of them by lions) [48]. High juvenile mortality in wild cheetahs, therefore, cannot be attributed to low genetic variation alone. The Florida panther is a second terrestrial example. Canonical panthers had lower heterozygosity than admixed panthers, which include offspring and backcrosses of the translocated Texas pumas [49]. Canonical panthers also had a much higher prevalence of cryptorchidism, kinked tails, and other traits historically associated with inbreeding depression [49].

Reproductive effects have also been examined in marine mammals, and the outcomes again differ between species. In wild bottlenose dolphins in Shark Bay, females that were inbred or had inbred calves had lower calving success, and inbred calves were weaned at an older age [50]. In North Atlantic right whales, by contrast, inbreeding had only a slight, statistically unclear effect on female fecundity and did not explain most of the variance in reproductive success [51]. There was, however, evidence consistent with reduced viability of inbred fetuses [51]. In northern elephant seals, associations were found in both sexes. Inbreeding and loss-of-function load were associated with lower lifetime reproductive success in females, and loss-of-function alleles at male fertility loci with lower paternity success in males [52]. Taken together, these studies show that the reproductive effects of inbreeding differ among species.

### 4.2. Survival and Developmental Traits

Effects on survival and early development are well documented in several wild mammals. In Soay sheep, each 10% increase in *F*_ROH_ is associated with 60% lower odds of survival in lambs, and inbreeding depression weakens with age [20]. Another study of Soay sheep found evidence of inbreeding depression due to an individual’s own or its mother’s inbreeding across all nine traits examined [53]. Negative effects on survival and birth weight have also been reported in wild red deer [54,55]. In meerkats, inbreeding is associated with reduced pup mass, growth, and juvenile survival [56]. The Isle Royale wolves are another well-documented example. This island population was extensively inbred, and its severe inbreeding depression has been attributed to increased homozygosity of strongly deleterious recessive mutations [57]. The wolves also showed congenital bone deformities [58]. As the population declined, the inbred wolves showed low reproduction and survival [59]. These findings show how inbreeding can reduce individual viability and thereby contribute to demographic decline in small, isolated populations.

### 4.3. Disease and Immune Function

Populations that lose genetic variation may become more susceptible to pathogens, both through inbreeding depression and through reduced variation in immune genes, such as those of the major histocompatibility complex (MHC) [60]. However, evidence that reduced MHC variation lowers population survival remains equivocal and difficult to disentangle from the effects of general inbreeding [60]. In striped dolphins, heterozygosity was strongly associated with lungworm load in females but not in males [61]. In California sea lions, sick animals had higher-than-normal parental relatedness, which varied among disease classes [62]. In wild red deer, inbreeding reduced juvenile survival, partly through strongyle nematode infection, providing evidence of parasite-mediated inbreeding depression [54]. Parasite-mediated selection against inbred individuals has also been reported in Soay sheep [63]. Genomic data alone can also flag populations for closer study. Compared with the giant otter, southern and northern sea otter genomes show very low genomic diversity, signs of recent inbreeding, and more putatively deleterious variants [64]. Associations between inbreeding and disease have therefore been reported in several wild mammals, but most are correlational.

## 5. Variation in Inbreeding Outcomes Across Mammalian Populations

Inbreeding outcomes vary because demographic history, mutation load, purging, and environmental context together determine how much deleterious variation a population carries and how much of it is expressed.

### 5.1. Demographic History and Population Bottlenecks

The genomic consequences of population decline differ among species. Genome sequences of three type D killer whales showed some of the highest inbreeding levels reported in mammals (*F*_ROH_ ≥ 0.65) and the lowest effective population size estimated from any killer whale genome [16]. The fitness consequences of this autozygosity have not been reported. In Indian tigers, a small, isolated population had higher inbreeding than large populations and the lowest loss-of-function load, consistent with purging, but also the highest predicted inbreeding depression [65]. Northern elephant seals provide a striking contrast. After near-extinction, they carry the genomic signature of an extreme bottleneck (estimated *N*_e_ = 6). Yet, one study found no evidence of inbreeding depression in body mass, blubber thickness, or susceptibility to parasites and disease [25]. The authors suggested that this may reflect purging of much of the genetic load during the bottleneck [25]. Another study, however, found that inbreeding and loss-of-function alleles were associated with lower reproductive success [52]. The two studies examined different traits, samples, and analytical approaches. Together they show that the absence of detectable inbreeding depression in some traits does not imply its absence in others. In wild Chinese rhesus macaques, deleterious homozygotes are enriched in long ROH, indicating that inbreeding is the main driver of the burden of homozygous deleterious alleles [66]. However, homozygous loss-of-function variants in the same macaques show evidence of purging [66]. Recovery is also possible. Whole-genome data from 418 koalas showed that historically bottlenecked but recovering populations had reduced mutational load, increasing *N*_e_ and newly regenerated rare variants [67]. The authors suggested that this pattern reflects rapid demographic expansion and that the reduced load in Victoria reflects stochastic loss during the bottleneck rather than systematic purging [67].

### 5.2. Genetic Purging

Purging can reduce the frequency of strongly deleterious recessive alleles, but it can coincide with the accumulation of mildly deleterious variants [27,36]. In Amur tigers, wild individuals are more inbred (*F*_ROH_ ≈ 0.50) than captive individuals (*F*_ROH_ ≈ 0.24) but carry fewer loss-of-function mutations, which suggests purging of large-effect alleles [68]. However, a higher ratio of homozygous mutation load and more fixed damaging alleles in the wild population indicate less efficient purging [68]. In Alpine ibex, severe bottlenecks appear to have purged highly deleterious mutations while mildly deleterious mutations accumulated [36]. Island foxes have exceptionally low diversity but have persisted for thousands of generations without the congenital defects common to other small and inbred populations [15]. This has been attributed to purging of strongly deleterious recessive alleles during long-term small population size [15]. The claim that island foxes show no inbreeding depression has also been challenged because they carry high levels of putatively harmful alleles, have low fecundity, and are susceptible to diseases that differ from island to island [69]. Genomic evidence of purging, such as a reduced burden of loss-of-function or highly deleterious mutations, should be distinguished from low genetic diversity without obvious inbreeding depression. The latter may reflect relatively benign conditions, under which inbreeding depression tends to be weaker [70]. It may also reflect low statistical power, meaning too few individuals or too little variation in inbreeding to detect an effect that is present [37]. A population can show genomic signatures of purging and still suffer strong inbreeding depression, as Southern Resident killer whales do for survival [71].

### 5.3. Connectivity and Fragmentation

Loss of connectivity affects mammals on land and at sea. On land, the fragmented Fennoscandian Arctic fox population shows longer ROH, signatures of recent inbreeding, and more homozygous deleterious mutations than the Siberian population [4]. In polar bears sampled in the Svalbard archipelago, there was a 3–10% loss of genetic diversity and a nearly 200% increase in genetic differentiation, which may best be explained by reduced gene flow as sea-ice cover declined [3]. In a dugong genome, ROH indicate past inbreeding, and the decline after the last interglacial period was likely driven by fragmentation and loss of seagrass habitat as sea levels fell [72]. Inshore bottlenose dolphin lineages generally have lower genomic diversity than offshore lineages [73]. Behaviour does not always prevent inbreeding either. In a free-ranging koala population, females did not avoid more closely related males [74]. In Shark Bay bottlenose dolphins, inbreeding occurs more often than expected by chance [50]. Together, these studies show that loss of connectivity, on land and at sea, reduces gene flow and exposes small populations to inbreeding. Maintaining or restoring connectivity is therefore a central management goal.

### 5.4. Environmental Modulation of Inbreeding Depression

The magnitude of inbreeding depression is not fixed. It can vary with environmental conditions [7]. Experimental studies and meta-analyses indicate that inbreeding depression is frequently, although not invariably, greater under stressful conditions [70,75]. The fitness benefits of gene flow into inbred populations are also larger in stressful than in benign environments [76]. A population may therefore show modest inbreeding depression under benign conditions but more pronounced effects under stress. Estimates obtained under benign conditions may thus underestimate its effects. In Soay sheep, for example, the greatest susceptibility of inbred individuals to parasitism occurred among adults at high population density [63]. Evidence from wild mammals is nevertheless mixed. In another study of Soay sheep, six of the nine traits showed inbreeding depression that tended to become stronger with increasing population density, although only two of these interactions were statistically significant [53]. Only two of these six patterns were statistically significant. When many tests are run, some results can look significant by chance, so a correction for multiple testing is applied. After this correction for the 14 tests performed, neither of the two significant results would remain significant [53].

### 5.5. Linking Genomic Inbreeding to Fitness: Association, Causation, and Confounding

Most evidence linking genomic inbreeding to fitness in wild mammals is correlational. An association between *F*_ROH_ and a fitness trait does not, by itself, show a direct causal effect of autozygosity because inbreeding can vary alongside other factors. In human case–control studies, for example, ROH–trait associations may be confounded by socioeconomic or cultural factors [11]. In wild populations, several factors can confound this link. They include population and family structure [41], population density, which can interact with inbreeding, and maternal inbreeding, which can, in turn, reduce offspring traits [53]. Parasites can also mediate inbreeding depression [54], so parasite burden is another factor to consider. Habitat quality, nutrition, age, cohort, and demographic history should also be considered when designing studies. Several approaches reduce these risks. Linear mixed models perform better when population or family structure is present [41]. Some studies also test whether fitness effects can be attributed to specific genomic regions [20,55]. Experimental or management interventions are especially informative when feasible. In Soay sheep, the survival disadvantage of more inbred individuals disappeared among sheep whose gastrointestinal parasites had been experimentally removed with anthelmintic treatment, indicating that parasites mediated the effect [63]. Failing to detect an association does not show that inbreeding depression is absent. Statistical power depends on how much inbreeding varies in the population, how accurately markers capture it, the size of the effect, and the number of individuals sampled [37]. Likewise, differences in mean *F*_ROH_ between species cannot be interpreted as differences in inbreeding depression without comparable fitness data.

Taken together, these examples show why similar levels of autozygosity can be associated with very different fitness outcomes (Table 1).

## 6. Conservation Implications and Genetic Rescue

Conservation programs often concentrate on extrinsic threats, and the effects of inbreeding depression on population dynamics are seldom measured in the wild [71]. In the endangered Southern Resident killer whales, for example, population models suggest that inbreeding depression constrains population growth and predicts further decline if the population stays genetically isolated and typical environmental conditions persist [71].

Genetic rescue has frequently increased the fitness of small, inbred populations [77]. A meta-analysis of gene flow into such populations across invertebrates, vertebrates, and plants found beneficial effects in 92.9% of 156 cases, with a low risk of outbreeding depression [76]. In the Florida panther, for example, numbers increased threefold, heterozygosity doubled, and survival and fitness measures improved after eight female pumas from Texas were translocated in 1995 [49]. Genetic rescue must, however, be distinguished from demographic rescue because a translocation may increase population growth simply by adding individuals. Genetic rescue has been defined as a rise in population fitness that exceeds what can be explained by the demographic contribution of immigrants alone [77]. Therefore, evidence for it ideally comes from designs that separate genetic effects from demographic and environmental effects. One example is a comparison among coexisting individuals with different amounts of immigrant ancestry. In bighorn sheep, reproduction, survival, and five fitness-related traits improved among descendants of 15 recent migrants, and trait values increased by 23–257% in maximally outbred individuals [78].

Genetic rescue is not free of risk, and it does not appear to be a universal solution [26]. One risk is outbreeding depression. Outbreeding depression is more likely that when source and recipient populations are distinct species, have fixed chromosomal differences, have had no gene flow for 500 years or more, or live in different environments [79]. Migrants also introduce both harmful and beneficial variants, and the magnitude and duration of the benefits are not yet well understood [80]. At Isle Royale, the benefit of genetic rescue appeared to wane after about two to three generations because of inbreeding, and the population declined as inbred wolves reproduced and survived poorly [59]. This case has divided opinion on how source populations should be chosen. Kyriazis et al. [35] cite the Isle Royale case as a reason for caution and propose drawing migrants from small or moderate-sized populations rather than large ones. Ralls et al. [69] argue that the arrival of a single unplanned male was not sound genetic rescue and that the evidence favors maximizing genetic diversity over minimizing the introduction of putatively harmful variants.

Terrestrial and marine species differ in the management options available to them. In terrestrial species, genetic rescue has been achieved by translocating individuals, as in the Florida panther [49]. In our view, direct translocation is likely to be impractical for most marine mammals, many of which live in socially structured populations. For many marine taxa, genetic management must therefore be largely indirect. It should focus on reducing human-caused mortality so that populations can grow, and on maintaining connectivity so that natural dispersal and gene flow can occur. For the vaquita, genome-informed simulations indicate that the population could recover if deaths stopped immediately [24]. Genetic diversity can also begin to recover through rapid demographic growth, as in koalas [67]. Management should not, however, aim to purge deleterious mutations by allowing continued inbreeding. Deliberate purging carries considerable short- and medium-term risks because an early fitness decline usually occurs before purging takes effect [26]. In computer simulations of conservation programs, inbred matings never led to higher fitness in the scenarios analyzed, and they usually retained less genetic diversity than random or minimum coancestry matings [81].

## 7. Future Directions

Future research should more directly link genomic inbreeding measures to fitness. It should combine ROH-based measures with information on specific deleterious variants, demographic history, trait data, and environmental context. Long-term studies are also needed to show how inbreeding effects vary across life stages and under changing ecological conditions. ROH-based inbreeding is negatively associated with fitness across taxa. Still, the estimated effect sizes are significantly influenced by ROH detection methodology, and there is an urgent need for standardized protocols [30]. IDrisk, which uses long ROH together with heterozygosity in non-ROH regions, has been proposed to predict the risk of inbreeding depression [29]. Such tools will need empirical validation across species. In Chinook salmon, inbred fish showed altered methylation at three of 22 genes examined [82]. To our knowledge, studies that directly link epigenetics, genomic inbreeding, and fitness in wild mammals remain scarce. Epigenetic analyses may eventually complement genomic approaches, but current evidence is too limited for them to serve as a primary framework for understanding inbreeding depression in wild mammals.

## 8. Discussion

Inbreeding depression in wild mammals is best understood as the result of interacting genomic, demographic, and ecological processes, rather than as a uniform consequence of small population size. Genetic factors, including inbreeding depression and deleterious mutations, contribute to extinction risk [5,83,84], yet they often receive less attention than extrinsic threats [71]. A central message of this review is that the path from inbreeding (a process) to autozygosity (its genomic consequence) to inbreeding depression (a fitness outcome) has several links that can weaken or break. Low genetic diversity should not be read as evidence of recent inbreeding. Below, we address four questions that arise from the case studies reviewed.

Why does *F*_ROH_ strongly predict fitness in some populations but not others? First, the fitness effect of a given amount of autozygosity depends on the masked load carried into it. Simulations suggest that large populations that contract face a substantially higher risk of extinction because inbreeding exposes strongly deleterious recessive variants [35]. Populations with a long history of small size [15] or that have passed through an extreme bottleneck [25] may carry less of this load. Second, ROH length matters. Most inbreeding in Scandinavian wolves was due to ancestors within the last 10 generations [43], and deleterious homozygotes in rhesus macaques were enriched in long ROH [66]. Third, detection depends on statistical power and study design. Survival traits show the greatest sensitivity to ROH across taxa [30], and inbreeding depression in Soay sheep decreases with age [20]. Inbreeding depression may also interact with environmental conditions, such as population density, although in the wild these interactions have been weak and difficult to detect [53]. Fourth, ROH-detection methodology significantly influences estimated effect sizes [30].

Why can populations with similar genome-wide diversity have very different fitness outcomes? Genome-wide diversity can be a poor guide. Inbreeding depression arises mainly when recessive deleterious mutations become homozygous through inbreeding, and in simulations, the number of strongly deleterious alleles, rather than heterozygosity, predicted extinction risk after genetic rescue [35]. Island foxes, for example, have very low diversity but, according to simulations, fewer strongly deleterious recessive alleles [15]. The vaquita carries few segregating deleterious variants [24]. Isle Royale wolves and Southern Resident killer whales, in contrast, show increased homozygosity of putatively deleterious variants together with evidence of fitness costs [57,71]. Environmental stress can also make inbreeding depression more severe [70], and parasites can mediate it [54]. Genetic variation remains fundamental to population fitness and adaptation to environmental change [1]. Even so, low diversity alone is not a reliable predictor of inbreeding depression.

When should managers consider intervention? Genomic data are most useful for management when combined with demographic and ecological information. On current evidence, we propose that intervention to increase gene flow should be considered when four conditions are met. First, the population is small and isolated, with little prospect of natural immigration. Second, individual inbreeding is high and increasing, particularly when much of it lies in long ROH. Third, fitness data, where available, show associations between inbreeding and survival or reproduction, or population growth is lower than expected given extrinsic conditions. Fourth, a suitable source population with a low predicted risk of outbreeding depression [79] is available. Where fitness data are unavailable, genetic load can be estimated from genome sequences [17]. IDrisk, which combines long ROH with heterozygosity outside ROH, offers a way to estimate the risk of inbreeding depression in a population [29]. ROH-based analyses covering the full range of ROH lengths are also promising monitoring tools [30]. Such indicators should be treated as signs of risk rather than as proof of inbreeding depression. Where translocation is not feasible, as is likely for many marine mammals, we suggest that reducing human-caused mortality should be the priority.

What evidence is currently missing? We see five main gaps. First, long-term, individual-based fitness data are needed for most threatened mammals, especially marine species. Second, studies should relate genomic estimates of realized and masked load to measured fitness because these estimates can be made without direct fitness data [17]. Third, standardized ROH detection protocols are needed [30]. Fourth, inbreeding depression with environmental interactions needs to be tested in natural populations [53]. Fifth, genetic rescue attempts should be monitored and replicated. This would allow tests of fitness effects across generations, separating gene flow effects from confounding factors [77]. It would also help to resolve the magnitude and duration of the benefits [80].

Inbreeding, therefore, remains an important concern for conservation management because it is a genetic extinction factor [12]. Small, inbred populations without gene flow are likely to be extirpated [76].

This review has limitations. It is a structured narrative review and is not exhaustive. The exact search strings were not recorded; a single author screened titles and abstracts; record-level screening numbers were not logged; and we did not formally assess study quality or carry out a meta-analysis. The case studies are weighted towards well-studied and charismatic species and towards published associations, so publication bias cannot be excluded. Differences in genomic data and analytical methods also prevented a direct quantitative comparison of studies.

Predicting extinction risk and guiding effective conservation action will require a more integrated framework that links genomic autozygosity, mutation load, life-history traits, and environmental stress.

## 9. Conclusions

Inbreeding depression is a major but highly variable force affecting the fitness and persistence of wild mammal populations. Genomic tools, especially ROH-based analyses, have greatly improved our ability to measure inbreeding and interpret its consequences. Genomic inbreeding, low diversity, and inbreeding depression are distinct, and high autozygosity alone does not demonstrate fitness costs. The same *F*_ROH_ value can have different consequences in different species, depending on mutation load, demographic history, life history, environmental conditions, and methodology. Universal genomic thresholds, therefore, cannot yet be applied across taxa. In the future, threat-assessment frameworks (such as those of the International Union for Conservation of Nature, IUCN) could consider genomic indicators of inbreeding and mutation load as complementary, taxon-specific information alongside census and effective population size. This should happen only once such indicators have been validated against fitness and population trajectories. In the meantime, genomic inbreeding metrics are best used to identify populations for closer monitoring and, together with demographic and ecological data, to inform decisions about gene flow and genetic rescue.

## Figures and Tables

**Table 1:** Summary of selected mammalian case studies evaluating genomic inbreeding, fitness consequences, and conservation implications. Genomic metrics were derived using different data types and methods and should be compared with caution. ROH, runs of homozygosity; *F*_ROH_, genomic inbreeding coefficient based on ROH; SNP, single-nucleotide polymorphism; IBD, identical by descent.

Species	Demographic History	Genomic Inbreeding Metric/Architecture	Observed Fitness Consequences	Purging and Conservation Implications	References
Florida panther (*Puma concolor coryi*)	Remnant population in southern Florida; eight female pumas from Texas were translocated in 1995 to increase depleted genetic diversity.	Average microsatellite heterozygosity lower in canonical Florida panthers than in admixed panthers.	Higher prevalence of cryptorchidism, kinked tails and thoracic cowlicks in canonical than in admixed panthers.	Following genetic restoration, panther numbers tripled and heterozygosity doubled; survival and other fitness measures improved, and inbreeding correlates declined.	[49]
Isle Royale wolf (*Canis lupus*)	Small, isolated island population [58] that later declined to the threshold of extinction [57].	Extensive inbreeding [57,58,59]; increased homozygosity of strongly deleterious recessive mutations [57].	Congenital malformations in the lumbosacral region in 58% of wolves examined [58]; low reproduction and survival as the population declined [59].	Severe inbreeding depression attributed to strongly deleterious recessive mutations [57]; the fitness benefit of genetic rescue appeared to wane after approximately 2–3 generations [59].	[57,58,59]
Type D killer whale (*Orcinus orca*)	Long-term small population size; lowest effective population size estimated from any killer whale genome.	Among the highest inbreeding levels reported in mammals (*F*_ROH_ = 0.65–0.73; three modern genomes).	No fitness data reported.	Fitness consequences of this autozygosity unknown.	[16]
Northern elephant seal (*Mirounga angustirostris*)	Near-extinction event; genomic signature of a recent extreme bottleneck (estimated *N*_e_ = 6) [25].	*F*_ROH_ ≈ 0.21; lower inbreeding load but higher realized (drift) load than the southern elephant seal [25]; increased frequency of loss-of-function alleles [52].	Inbreeding and loss-of-function load associated with lower female lifetime reproductive success, and loss-of-function alleles at male-fertility loci with lower paternity success [52]; no evidence of inbreeding depression in body mass, blubber thickness or susceptibility to parasites and disease [25].	The bottleneck may have purged much of the genetic load, which could explain the lack of inbreeding depression in the traits studied [25]. Loss-of-function alleles retained after the bottleneck were associated with lower reproductive success [52]. The two studies examined different traits.	[25,52]
Soay sheep (*Ovis aries*)	Individually monitored population on St Kilda [20,53] (5952 sheep genotyped [20]).	Mean *F*_ROH_ ≈ 0.24 (range 0.18–0.50) [20]; genomic inbreeding estimated from 37,037 SNPs in another study [53].	Reduced survival, with inbreeding depression decreasing with age (a 10% increase in *F*_ROH_ associated with a 60% reduction in the odds of lamb survival) [20]; some evidence of inbreeding depression in all nine traits examined in another study [53].	Links genomic inbreeding to survival across the lifespan and identifies contributing genomic regions [20].	[20,53]
Island fox (*Urocyon littoralis*)	Persisted for thousands of generations and remained at small size with low diversity for many generations [15].	Exceptionally low diversity [15]; elevated homozygosity of putatively deleterious alleles [15], but simulations suggest a reduced burden of strongly deleterious recessive alleles [15].	Congenital skeletal defects rare (7 of 119 specimens) and not more frequent than in mainland gray foxes [15].	Interpreted as purging of strongly deleterious recessive alleles [15]; this interpretation has been disputed [69].	[15,69]
Southern resident killer whale (*Orcinus orca*)	Endangered; predicted to decline further if the population remains genetically isolated and typical environmental conditions continue.	More inferred homozygous deleterious alleles than three other, growing populations.	Strong inbreeding depression for survival; population models suggest that inbreeding depression limits population growth.	Inbreeding depression despite genomic signatures of purging; genetic factors are often neglected relative to extrinsic threats.	[71]
Vaquita (*Phocoena sinus*)	Historically rare; declined to ~10 individuals because of bycatch in gillnets.	Low burden of segregating deleterious variation (20 genomes).	No direct fitness data reported.	Historical rarity is inferred to have reduced the risk of inbreeding depression; recovery depends on halting bycatch mortality.	[24]

## Data Availability

All data discussed are available in the published studies cited herein. No new data were created or analysed in this study. Data sharing does not apply to this article.

## Supplementary Materials

The following supporting information can be downloaded at: https://www.mdpi.com/article/10.3390/ani16203268/s1, Table S1. Primary empirical studies of inbreeding, genomic diversity and fitness in wild (or managed) mammal populations discussed in the review, grouped by taxonomic group. Reference numbers refer to the revised reference list of the main text.

## Abbreviations

The following abbreviations are used in this manuscript:

ROH	Runs of homozygosity
SNP	Single-nucleotide polymorphism
MHC	Major histocompatibility complex
*F*_ROH_	Genomic inbreeding coefficient based on runs of homozygosity
*F*_p_	Pedigree-based inbreeding coefficient
IBD	Identical by descent
IBS	Identical by state
*N*_e_	Effective population size
IUCN	International Union for Conservation of Nature
PRISMA	Preferred Reporting Items for Systematic Reviews and Meta-Analyses
