# Supplementary material for: Inbreeding Depression in Wild Mammals: Genomic Mechanisms and Conservation Consequences
Source: Animals (Basel). 2026 Oct 16;16(20):3268. doi: 10.3390/ani16203268 (PMC13700819; doi:10.3390/ani16203268)
Supplement: Supplementary file 1 [file animals-16-03268-s001.zip › animals-4597552-supplementary.pdf]

Supplementary Materials

Inbreeding Depression in Wild Mammals: Genomic Mechanisms and Conservation Consequences. Anwar, A.; Chung, T.Y.T.; Zhu, C.; Tsui, H.C.L.; Kot, B.C.W.

Table S1. Primary empirical studies of inbreeding, genomic diversity and fitness in wild (or managed) mammal populations discussed in the review, grouped by taxonomic group. Reference numbers refer to the revised reference list of the main text.

Group	Species / population	Ref.	Genetic/genomic measure	Fitness trait(s) or outcome	Main finding as used in the review
Carnivora (terrestrial)	South African cheetah (*Acinonyx jubatus jubatus*)	[47]	Allozymes; MHC variation via reciprocal skin grafts	Captive breeding, juvenile mortality (captivity and wild), spermatozoal abnormalities	Extreme paucity of genetic variation (probably a recent bottleneck), extending to the MHC; these traits described as apparent consequences
Carnivora (terrestrial)	Cheetah (*Acinonyx jubatus*)	[48]	— (ecological field study)	Juvenile mortality in the wild	Extremely high juvenile mortality; lion predation a major source
Carnivora (terrestrial)	Florida panther	[49]	Heterozygosity; genetic restoration (eight Texas pumas translocated in 1995)	Population size, survival, fitness measures	Numbers increased threefold, heterozygosity doubled, survival and fitness measures improved
Carnivora (terrestrial)	Grey wolf, Isle Royale (*Canis lupus*)	[59]	Genetic ancestry/pedigree	Reproduction, survival, population trajectory	Benefit of genetic rescue waned after ~2–3 generations because of inbreeding
Carnivora (terrestrial)	Grey wolf, Isle Royale	[57]	Whole genomes (11 Isle Royale wolves plus comparison wolves); ROH; deleterious variants; simulations	Vertebral (skeletal) malformations	Extensive inbreeding; severe ID attributed to homozygosity of strongly deleterious recessive mutations
Carnivora (terrestrial)	Grey wolf, Isle Royale	[58]	— (morphology in a known inbred population)	Congenital bone deformities	Lumbosacral malformations in 58% (n = 36); lumbosacral transitional vertebrae in 33% vs. 1% (1/99) in two outbred populations
Carnivora (terrestrial)	Grey wolf, Scandinavia	[43]	Whole-genome sequencing; F_{ROH} vs. F_P	— (genomic; F_P vs. genomic estimators compared)	F_P strongly correlated with genomic inbreeding (r^2 = 0.86); most inbreeding from long IBD segments
Carnivora (terrestrial)	Island fox (*Urocyon littoralis*)	[15]	Genomes; simulations	Morphological (congenital defects)	No morphological evidence of inbreeding depression; simulations support reduced strongly deleterious load
Carnivora (terrestrial)	Tiger, India (*Panthera tigris*)	[65]	Whole genomes; ROH; mutation load	Predicted inbreeding depression	Small isolated population most inbred, with the lowest loss-of-function load but the highest predicted inbreeding depression
Carnivora (terrestrial)	Amur tiger (*P. t. altaica*)	[68]	Whole genomes; F_{ROH}; mutation load	— (genomic)	Wild more inbred than captive; fewer loss-of-function but more fixed damaging alleles
Carnivora (terrestrial)	Arctic fox (*Vulpes lagopus*)	[4]	Genomes; ROH; deleterious variants	— (genomic)	Longer ROH and more homozygous deleterious mutations in northern Fennoscandia than in Siberia
Carnivora (sea-ice-dependent)	Polar bear (*Ursus maritimus*), Barents Sea	[3]	22 microsatellite loci, 626 individuals	— (population genetic)	3–10% loss of diversity and ~200% increase in differentiation, which may best be explained by reduced gene flow from sea-ice-related

Group	Species / population	Ref.	Genetic/genomic measure	Fitness trait(s) or outcome	Main finding as used in the review
					fragmentation
Carnivora (terrestrial)	Meerkat (*Suricata suricatta*)	[56]	Pedigree (F_P)	Pup mass, growth, hind-foot length, juvenile survival	Inbreeding depression in several early-life traits
Artiodactyla (ungulates)	Soay sheep (*Ovis aries*)	[20]	417K imputed SNPs; F_{ROH}	Annual survival	10% increase in F_{ROH} → 60% lower odds of lamb survival; effect declines with age
Artiodactyla (ungulates)	Soay sheep, St Kilda	[53]	Genomic inbreeding coefficients (37,037 SNPs)	Nine traits × population density	Some evidence of ID (own or maternal inbreeding) in all traits; only 2 of 14 ID × density tests significant at $P < 0.05$, none after Bonferroni correction
Artiodactyla (ungulates)	Soay sheep	[63]	Microsatellite heterozygosity	Parasite load, survival	Parasite-mediated selection against inbred individuals
Artiodactyla (ungulates)	Red deer (*Cervus elaphus*)	[19]	37,410 SNPs (genomic coefficient) vs. pedigree (F_P)	Birth weight, juvenile survival, adult annual survival and breeding success, lifetime breeding success	Genomic, but not pedigree, coefficients detected ID in adult traits (annual breeding success)
Artiodactyla (ungulates)	Red deer	[55]	SNPs; ROH-based, chromosome-specific	Birth weight, survival, lifetime breeding success	ID likely results from many mildly/moderately deleterious mutations across chromosomes (no major-effect loci detected)
Artiodactyla (ungulates)	Red deer	[54]	Genomic inbreeding	Juvenile birth weight and overwinter survival; helminth parasite burden; adult female overwinter survival and fecundity	Parasite (strongyle)-mediated inbreeding depression
Artiodactyla (ungulates)	Bighorn sheep (*Ovis canadensis*)	[78]	Immigrant ancestry	Reproduction, survival, five fitness-related traits	Traits 23–257% higher in maximally outbred descendants of migrants
Artiodactyla (ungulates)	Scimitar-horned oryx (*Oryx dammah*)	[18]	Whole genomes; ROH; deleterious variants	— (genomic)	Unmanaged groups more inbred with higher homozygous load than managed groups
Artiodactyla (ungulates)	Alpine ibex (*Capra ibex*)	[36]	Whole genomes; deleterious variants	— (genomic)	Purging of highly deleterious and accumulation of mildly deleterious mutations
Primates	Wild Chinese rhesus macaque (*Macaca mulatta*)	[66]	79 whole genomes; ROH; deleterious variants	— (genomic)	Deleterious homozygotes enriched in long ROH; evidence that homozygous LoF variants are being purged
Diprotodontia (marsupials)	Koala (*Phascolarctos cinereus*)	[74]	DArTseq SNPs (pairwise and internal relatedness; parentage)	Mate choice, disease avoidance	No avoidance of related or Chlamydia-infected males
Diprotodontia (marsupials)	Koala	[67]	Whole-genome data, 418 koalas	— (genomic)	Recovering populations: reduced mutational load, increasing Ne, new rare variants
Cetacea	Killer whale, Southern Residents (*Orcinus orca*)	[71]	Whole genomes; F_{ROH}; deleterious variants	Survival; population growth (models)	Strong ID for survival; ID predicted to limit growth
Cetacea	Killer whale, type D	[16]	Whole genomes of 3 modern individuals plus low-coverage genomic data from 1 museum	— (fitness not assessed)	$F_{ROH} \geq 0.65$, among the highest in mammals

Group	Species / population	Ref.	Genetic/genomic measure	Fitness trait(s) or outcome	Main finding as used in the review
			specimen; F_{ROH} (ROH > 1.5 Mb) from the 3 modern genomes		
Cetacea	Killer whale (global)	[44]	Genomes; ROH	— (demographic history)	Short ROH reflect deep coalescence; longer, younger ROH in populations of conservation concern
Cetacea	Bottlenose dolphin (*Tursiops* sp.), East Shark Bay	[50]	16 microsatellites; internal relatedness	Calving success, weaning age	Inbreeding higher than expected by chance; inbred females and, independently, females with inbred calves had lower calving success; inbred calves weaned later
Cetacea	North Atlantic right whale (*Eubalaena glacialis*)	[51]	ddRAD-seq (~2% of genome), 105 females; several genomic inbreeding coefficients	Female reproductive success	Only a slight negative effect of inbreeding on female fecundity (not the main driver of variance); evidence consistent with reduced viability of inbred fetuses
Cetacea	Striped dolphin (*Stenella coeruleoalba*)	[61]	43,018 RAD SNPs; heterozygosity	Lungworm burden	Heterozygosity–parasite load association in females only
Cetacea	Vaquita (*Phocoena sinus*)	[24]	20 genomes; simulations	— (simulated extinction risk)	Low deleterious burden; recovery predicted if bycatch ceases
Pinnipedia	Northern elephant seal (*Mirounga angustirostris*)	[25]	22 microsatellites and 15,051 RAD SNPs (ID tests); whole-genome resequencing; demographic modelling; simulations	Body mass, blubber, parasites, disease	No evidence of ID in these traits; bottleneck $N_e = 6$; purging of much of the genetic load inferred
Pinnipedia	Northern elephant seal	[52]	Genomes	Fitness of breeding males and females	Inbreeding, LoF alleles and allele-frequency distortion associated with reduced fitness
Pinnipedia	California sea lion (*Zalophus californianus*)	[62]	Genetic estimate of parental relatedness	Disease status	Sick animals had higher parental relatedness
Carnivora (marine)	Sea otter (*Enhydra lutris*)	[64]	Genomes	— (genomic)	Extremely low genomic diversity; signals of recent inbreeding; more putatively deleterious variants
Sirenia	Dugong (*Dugong dugon*), one Moreton Bay individual	[72]	Reference genome (single individual); heterozygosity; ROH; demographic inference	— (genome assembly; demographic history)	Relatively high heterozygosity; no recent inbreeding, but ROH indicate some past inbreeding; decline after last interglacial

Summary: Carnivora (terrestrial): 12; Carnivora (sea-ice-dependent): 1; Artiodactyla (ungulates): 9; Primates: 1; Diprotodontia (marsupials): 2; Cetacea: 7; Pinnipedia: 3; Carnivora (marine): 1; Sirenia: 1. Total: 37 studies.
